# Proto-oncogene mutations in middle ear cholesteatoma contribute to its pathogenesis

**DOI:** 10.1186/s12920-023-01640-6

**Published:** 2023-11-15

**Authors:** Chisei Satoh, Koh-ichiro Yoshiura, Hiroyuki Mishima, Haruo Yoshida, Haruo Takahashi, Yoshihiko Kumai

**Affiliations:** 1 Department of Otolaryngology-Head and Neck Surgery, Nagasaki University Graduate School of Biomedical Sciences, Nagasaki, Japan; 2 Department of Human Genetics, Nagasaki University Graduate School of Biomedical Sciences, Nagasaki, Japan; 3 Leading Medical Research Core Unit, Nagasaki University Graduate School of Biomedical Sciences, Nagasaki, Japan

**Keywords:** Cholesteatoma, Gene mutation, Notch 1, Exome analysis

## Abstract

**Background:**

Chronic inflammation causes bone destruction in middle ear cholesteatomas (MECs). However, the causes of their neoplastic features remain unknown. The present study demonstrated for the first time that neoplastic features of MEC are based on proto-oncogene mutations.

**Results:**

DNA was extracted from MEC and blood samples of five patients to detect somatic mutations using depth-depth exome sequencing. Exons with somatic variants were analyzed using an additional 17 MEC/blood test pairs. Variants detected in MECs but not in blood were considered pathogenic variant candidates. We analyzed the correlation between proto-oncogene (*NOTCH1* and *MYC*) variants and the presence of bone destruction and granulation tissue formation. *MYC* and *NOTCH1* variants were detected in two and five of the 22 samples, respectively. Two of the *NOTCH1* variants were located in its specific functional domain, one was truncating and the other was a splice donor site variant. Mutations of the two genes in attic cholesteatomas (n = 14) were significantly related with bone destruction (p = 0.0148) but not with granulation tissue formation (p = 0.399).

**Conclusions:**

This is the first study to demonstrate a relationship between neoplastic features of MEC and proto-oncogene mutations.

**Supplementary Information:**

The online version contains supplementary material available at 10.1186/s12920-023-01640-6.

## Background

Cholesteatomas are benign, squamous epithelial hyperproliferative conditions of the tympanic cavity associated with keratin debris accumulation [1]. They cause gradual destruction of temporal structures, including the ossicles, facial nerve canal, and skull base. This process may be accompanied by severe complications, including facial paralysis, meningitis, and intracranial abscesses [2]. These complications can only be avoided by timely surgical removal of the pathology. Hence, there is a need to establish preventive nonsurgical treatments based on the pathogenesis [2].

Various hypotheses regarding the origin and pathogenesis of cholesteatomas have been proposed [3, 4]. Cholesteatomas are fundamentally non-neoplastic lesions of the temporal bone, but are clinically similar to neoplasms because of their unique epithelial hyperproliferative nature [3]. Interestingly, chronic inflammation may contribute to the pathological aggressiveness by affecting the degree of epithelial migration, cell proliferation, and extracellular matrix deposition [3]. However, it remains unknown whether these neoplasm-like aggressive features are caused by genetic mutations. To develop a fundamental understanding of cholesteatomas without distal metastasis, it is important to investigate underlying genes or mutations, particularly proto-oncogenes, that may control cellular proliferation.

In the present study, we analyzed the pathological genetic variants that may be related to cholesteatomas and investigated whether somatic mutations were associated with bone destruction and inflammatory reactions, such as granulation tissue formation.

## Results

**Fig. 1:**
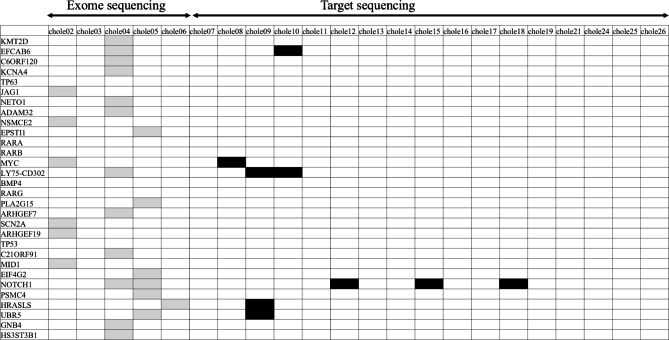
Gene variants detected in each cholesteatoma sample Samples 02–06 were analyzed via whole-exome sequencing, while samples 07–26 were analyzed using target capture sequencing. Genes with somatic genetic variants are listed. *MYC* variants were detected in two samples, while *NOTCH1* variants were detected in five samples. Gray and black boxes indicate genes with variants

**Fig. 2:**
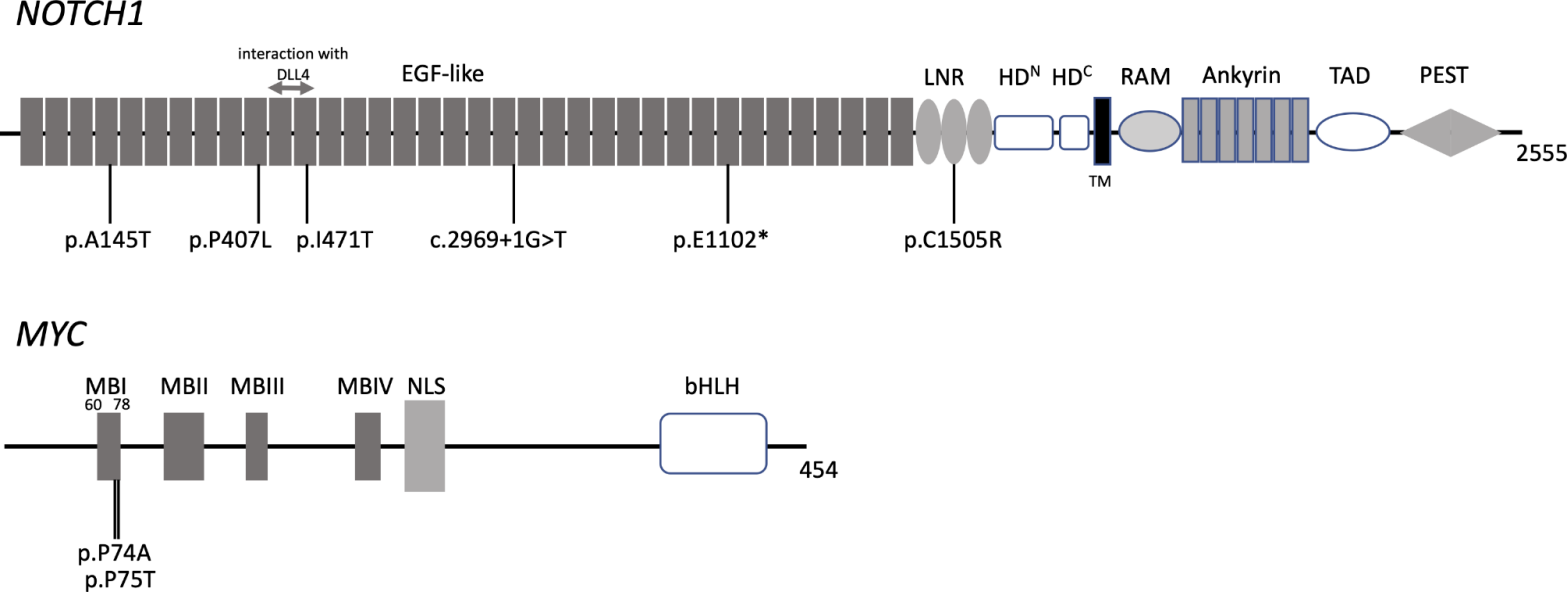
Domain graph for *NOTCH1* and *MYC* with locations of the detected variants One variant (p.I471T) was located in the 11th and 12th EGF repeat domains, while one (p.C1505R) was located in the LNR domain. EGF repeat and LNR domains were functionally significant in *NOTCH1*, and therefore, mutations in these domains could result in loss of *NOTCH1* function

As a result of deep-depth WES with 137–212 mean depth of coverage, 24 potential cholesteatoma pathogenic genes (25 somatic variants) were identified (Table 1). The genes with somatic genetic variants detected using WES (samples 02–06) and target capture sequencing (samples 07–19, 21, 24–26) are shown in Tables 1 and 2, respectively. Genes with mutations found in each sample are shown in Fig. 1. *MYC* and *NOTCH1* variants of interest were detected in two and five samples, respectively. Variants in *LY75-CD302*, *EFCAB6*, *HRASLS*, and *UBP* with approximately 50% variant allele frequencies (VAF) could be somatic variants acquired before cholesteatoma development. All of these variants are registered in the dbSNP database and may not be pathogenic. Meanwhile, the VAF for *NOTCH1* was 2–7%, indicating that somatic mutations accounted for a small portion of cholesteatomas. One of the variants (p.I471T) was located in the 11th and 12th epidermal growth factor (EGF) repeat domain, while another (p.C1505R) was located in the LIN-12/NOTCH repeats (LNR) domain. EGF repeat and LNR domains are functionally significant in *NOTCH1* [5], and therefore, mutations in these domains could result in the loss of *NOTCH1* function (Fig. 2). Variants c2969 + 1 G > T and p.E1102* were a splice alteration and nonsense mutation, respectively, indicating deleterious mutations. *NOTCH1* and *MYC* mutations correlated significantly with bone destruction (p = 0.0148) but not with granulation tissue formation (p = 0.399) (Supplement Fig. 1-a, b) in attic cholesteatomas (n = 14) (Table 3). Bone destruction was defined as at least one of the following clinical or surgical findings: ossicle destruction (> 50%) (Supplement Fig. 1-c, d), dura exposure, facial nerve exposure, and labyrinthine fistulae, described in operative note by surgeons.

**Table 1:** Somatic genetic variants detected using Exome sequencing

sample	Gene	Locus (hg19)	Reference allele	Alternative allele	Variant allele frequency	Function	Variant	Accession number
chole04	ADAM32	chr8:39027505–39,027,505;G > A	85	12	0.12	exonic	c.G904A:p.G302R	ENST00000519315.1
chole02	ARHGEF19	chr1:16534478–16,534,478;C > T	41	9	0.18	exonic	c.G655A:p.A219T	ENST00000270747.3
chole04	ARHGEF7	chr13:111920009–111,920,009;G > A	170	28	0.14	exonic	c.G235A:p.G79R	ENST00000544132.1
chole04	C21orf91	chr21:19190626–19,190,626;C > T	299	39	0.12	exonic	c.G10A:p.E4K	ENST00000400558.3
chole04	C6orf120	chr6:170103003–170,103,003;C > G	317	48	0.13	exonic	c.C448G:p.P150A	ENST00000332290.2
chole04	EFCAB6	chr22:44068144–44,068,144;G > C	257	33	0.11	exonic	c.C1461G:p.F487L	ENST00000262726.7
chole05	EIF4G2	chr11:10825526–10,825,526;A > T	344	41	0.11	exonic	c.T622A:p.L208M	ENST00000396525.2
chole05	EPSTI1	chr13:43462434–43,462,434;->TTAGG	190	38	0.17	exonic	c.1184_1185insCCTAA:p.E395fs	ENST00000313640.7
chole04	GNB4	chr3:179143948–179,143,948;A > C	149	20	0.12	exonic	c.T41G:p.L14R	ENST00000232564.3
chole06	HRASLS	chr3:192959038–192,959,038;G > T	0	2	1.00	exonic	c.G31T:p.A11S	ENST00000264735.2
chole04	HS3ST3B1	chr17:14248860–14,248,860;A > G	206	29	0.12	exonic	c.A1070G:p.H357R	ENST00000360954.2
chole02	JAG1	chr20:10639143–10,639,143;C > G	214	54	0.20	exonic	c.G667C:p.G223R	ENST00000254958.5
chole04	KCNA4	chr11:30033739–30,033,739;C > T	164	22	0.12	exonic	c.G487A:p.G163S	ENST00000328224.6
chole04	KMT2D	chr12:49421623–49,421,623;A>-	417	57	0.12	exonic	c.14606delT:p.L4869fs	ENST00000301067.7
chole04	LY75-LY75-CD302	chr2:160734945–160,734,945;T > A	368	57	0.13	exonic	c.A1664T:p.Y555F	ENST00000553424.1
chole02	MID1	chrX:10,417,566–10,417,566;C > T	251	43	0.15	exonic	c.G1846A:p.A616T	ENST00000453318.2
chole02	MYC	chr8:128750683–128,750,683;C > G	242	45	0.16	exonic	c.C220G:p.P74A	ENST00000377970.2
chole04	NETO1	chr18:70526220–70,526,220;G > A	338	38	0.10	exonic	c.C307T:p.R103X	ENST00000397929.1
chole04	NOTCH1	chr9:139402705–139,402,705;C > A	97	33	0.25	exonic	c.G3304T:p.E1102X	ENST00000277541.6
chole05	NOTCH1	chr9:139412233–139,412,233;A > G	187	49	0.21	exonic	c.T1412C:p.I471T	ENST00000277541.6
chole02	NSMCE2	chr8:126194499–126,194,499;->T	125	20	0.14	splicing	c.418 + 1->T	ENST00000287437.3
chole05	PLA2G15	chr16:68279402–68,279,402;A > G	306	39	0.11	exonic	c.A73G:p.M25V	ENST00000566188.1
chole05	PSMC4	chr19:40485876–40,485,876;G > A	419	59	0.12	exonic	c.G826A:p.D276N	ENST00000157812.2
chole02	SCN2A	chr2:166226663–166,226,663;C > T	138	37	0.21	exonic	c.C3703T:p.R1235X	ENST00000357398.3
chole05	UBR5	chr8:103307283–103,307,283;T > C	243	31	0.11	exonic	c.A4097G:p.N1366S	ENST00000521922.1

**Table 2:** Somatic genetic variants detected using target capture sequencing

Samples	Gene	Locus (hg19)	Reference allele count	Alternative allele count	Variant allele frequency	function	Variant	Accession number
chole10	LY75-LY75-CD302	chr2:160676343–160,676,343;C > T	533	511	0.489	exonic	c.G4047A:p.L1349L	ENST00000505052.1
chole09	LY75-LY75-CD302	chr2:160743040–160,743,040;T > A	254	258	0.504	exonic	c.A804T:p.E268D	ENST00000554112.1
chole09	LY75-LY75-CD302	chr2:160755347–160,755,347;C > T	359	335	0.483	exonic	c.G318A:p.L106L	ENST00000554112.1
chole10	EFCAB6	chr22:44131786–44,131,786;T > C	485	481	0.498	exonic	c.A139G:p.R47G	ENST00000396231.2
chole09	HRASLS	chr3:192959302–192,959,302;A > C	286	284	0.498	exonic	c.A295C:p.R99R	ENST00000264735.2
chole09	UBR5	chr8:103308010–103,308,010;T > C	236	281	0.544	exonic	c.A3666G:p.K1222K	ENST00000520539.1
chole08	MYC	chr8:128750686–128,750,686;C > A	950	28	0.029	exonic	c.C223A:p.P75T	ENST00000377970.2
chole18	NOTCH1	chr9:139399835–139,399,835;A > G	377	9	0.023	exonic	c.T4513C:p.C1505R	ENST00000277541.6
chole12	NOTCH1	chr9:139404184–139,404,184;C > A	891	40	0.043	splicing	c2969 + 1 G > T	ENST00000277541
chole15	NOTCH1	chr9:139412624–139,412,624;G > A	905	41	0.043	exonic	c.C1220T:p.P407L	ENST00000277541.6
chole15	NOTCH1	chr9:139417611–139,417,611;C > T	739	54	0.068	exonic	c.G433A:p.A145T	ENST00000277541.6

**Table 3:** Patient profiles showing presence or absence of clinical presentations and gene mutations

Patient	age	analysis	type	stage	ossicles destruction	Granulation	dura exposure	Facial nerve exposure	Labilynthin fistura	Facial palsy	variants
2	38	exome	PF	II	< 50%	+	+	–	–	–	myc
3	17	exome	PF	Ib	< 50	–	–	–	–	–	
4	57	exome	PF	II	> 50	+	+	+	–	–	Notch
5	73	exome	PT	II	> 50	–	–	+	–	–	Notch
6	54	exome	PF	II	< 50	–	–	–	–	–	
7	28	Taget capture	PF	II	< 50	+	–	–	–	–	
8	59	Taget capture	PF	III LF	< 50	+	+	+	+	–	Myc
9	49	Taget capture	PF	II	< 50	+	+	+	–	–	
10	41	Taget capture	PF	II	< 50	+	–	–	–	–	
11	49	Taget capture	R		> 50	+	+	–	–	–	
12	64	Taget capture	R		< 50	–	–	–	–	–	Notch
13	73	Taget capture	secondary	II	< 50	+	–	+	–	–	
14	29	Taget capture	PF	III LF	> 50	+	+	+	+	–	
15	64	Taget capture	PF	III LF	> 50	+	+	+	+	–	Notch
16	57	Taget capture	C	III FP	< 50	–	+	+	+	+	
17	60	Taget capture	secondary	II	< 50	+	–	–	–	–	
18	74	Taget capture	PT	Ia	< 50	–	–	–	–	–	Notch
19	33	Taget capture	PF	Ib	< 50	+	–	–	–	–	
21	56	Taget capture	PF	Ib	< 50	–	–	–	–	–	
24	5	Taget capture	PF	II	< 50	–	–	–	–	–	
25	17	Taget capture	R			–	–	–	–	–	
26	6	Taget capture	PF	II	< 50	+	–	–	–	–	

PF, pars flaccida (attic cholesteatoma); PT, pars tensa; R, recurrent; C, congenital; LF, labyrinthine fistula; FP, facial palsy

## Discussion

Acquired cholesteatomas are considered non-neoplastic pathologies with epithelial keratinizing lesions, which may lead to invasion and/or destruction of the temporal bone [2–4]. Intracranial complications caused by bony destruction can be fatal, therefore, preventive treatment prior to surgical removal of the lesion is necessary. Despite a number of previous studies, the origin of this pathology remains unclear [6]. Alternative molecular strategies, including exploration of genetic alterations, may expand the spectrum of therapeutic choices and lead to the development of nonsurgical preventive options for cholesteatomas. The present study used exome sequencing in five cholesteatoma patients to demonstrate somatic mutations in 24 genes. Gene Ontology (GO) analysis with these candidate genes (Supplement data), including *MYC*, *NOTCH1*, *JAG1*, and *PSMC4*, demonstrated significant correlations with mesenchymal transition, cell development, cell differentiation, and cellular response to hypoxia, which are clinically assumed to be involved in the pathology of the disease. Exon sequencing of these genes in 17 cholesteatoma/blood test pairs revealed that somatic variants in *MYC* and *NOTCH1* had the highest frequencies among the examined genes. Whole-exome sequencing (WES) analysis was performed on the first five specimens, and the presence of gene mutations in previously known neoplastic variants could be precisely evaluated at the whole-gene level. However, the remaining 17 samples were evaluated with target capture sequencing; these samples may have other mutations in protooncogenes, which are not included in the 24 specific genes already detected in the present study. Moreover, mutations of either gene in attic cholesteatomas were significantly related to bone destruction (p = 0.0148) but not with granulation tissue formation(p = 0.399).

A review of the potential proto-oncogenic modifications in cholesteatomas failed to provide sufficient genetic evidence to support neoplastic features [7]. However, some studies have demonstrated its high proliferative activity using a variety of proliferation markers, including cytokeratin 13/16, Ki67, and proliferating cell nuclear antigen [8–10]. Further genetic analysis is required to confirm the highly proliferative nature of the pathology [11]. Interestingly, recent studies using microarray analysis techniques have demonstrated that cholesteatomas express many tumor-related genes, including proto-oncogenes c-*MYC* and *NOTCH1* [11–15]. Based on these studies, abnormalities of proto-oncogene expression in cholesteatomas appear to be linked to their neoplastic features.

In agreement with previous studies, the present study found that bone destruction in cholesteatomas was significantly associated with proto-oncogene mutations. Furthermore, we also detected variants in the 11th and 12th EGF repeat domains and LNR domain, which have previously been shown to be functionally important for *NOTCH1* [5]. *NOTCH1* is expressed on the cell surface as a heterodimer composed of non-covalently associated extracellular (NEC) and transmembrane subunits. The NEC subunit consists of 36 iterated EGF-like repeats that include the binding region and three LNRs [16] where mutations were found in the present study. Extracellular domains of NOTCH receptors are largely composed of tandemly repeated EGF-1 domains. The 11th and 12th EGF repeat domains, in which the variant was detected in this study, were identified as necessary and sufficient to mediate binding [17]. The importance of these domains in Delta-Serrate-Lag2 ligand binding has been reported in multiple studies [18–20]. Our findings suggest an association between *NOTCH1* and neoplastic features of cholesteatomas [15]. A previous study reported significantly decreased *NOTCH1* expression in cholesteatoma epithelium compared to auditory canal skin epithelium, suggesting that *NOTCH1* may alter the balance from cellular differentiation to hyperproliferation and subsequently contribute to neoplastic features of the pathology [15]. In the present study, immunohistochemical analysis of *NOTCH1* was performed in a surgical sample (case not shown in Table I). In this specific sample, the levels of expression of *NOTCH1* and downstream *HES1* in the basement membrane of cholesteatoma epithelium were weaker than in normal skin (Supplement Fig. 2-a and -b). These observations suggest that there is a positive relation between *NOTCH1* mutation and protein expression in these lesions. Martincorena et al. [21] reported a high frequency (30–80%) of *NOTCH1* mutations in the esophageal epithelia of elderly and middle-aged healthy individuals, suggesting that it is a passenger, not a driver, mutation. That is, high VAFs, including *NOTCH1*, indicate that clonal expansion in the elderly may result in a predisposition to tumor formation and progression. VAF refers to the proportion of specific variants detected at a particular location in the genome relative to the number of sequencing reads; a value of 50% or 100% is taken to indicate a germline variant, while in the case of somatic mutations VAF varies based on the proportion of a cell population with the mutation within the extracted sample. In this study, VAF ranged from 2 to 8%, indicating a somatic mutation. Protooncogene variants may enhance the pathological features of benign conditions, including cholesteatomas. Therefore, we hypothesized that VAF in *NOTCH1* may be useful for identifying the clinical behavior of cholesteatomas, including the degrees of bony destruction.

Finally, we also found that the VAF of *LY75-CD302*, *EFCAB6*, *HRASLS*, and *UBP* were approximately 50% (Table 2; samples 9 and 10), indicating the presence of heterozygous gene variants. This suggests clonality of cholesteatomas that expanded from a small number of cells. However, this finding requires further investigation using larger samples for exome and targeted mutation analyses.

The present study had some limitations. First, the sample size of 22 was small, so further exome and targeted mutation analyses with larger sample sizes are needed to analyze the roles of genetic changes in cholesteatoma development, inflammation, and neoplastic features. Second, definitive identification of a tissue as neoplastic or whether protooncogene variants induce neoplasia, appropriate validation is required using nearby nonneoplastic tissues or homologous tissue biopsy specimens from the same patient, such as skin from the external auditory canal, in addition to blood samples. Such tissue controls would provide more robust data and confirmation to clarify the correlations between genetic mutations and clinical presentations of cholesteatoma.

## Conclusions

Mutations in cholesteatomas, including *NOTCH1* and *MYC*, were significantly correlated with bone destruction. These observations suggest that protooncogene mutations may enhance the pathological features of cholesteatomas.

### Methods

Blood and cholesteatoma samples were collected from five Japanese cholesteatoma patients who were treated surgically. These blood-cholesteatoma paired samples were subjected to whole-exome sequencing (WES). DNA was extracted from cholesteatoma using the QIAamp DNA Mini kit (QIAGEN, Hilden, Germany) and from blood using the QIAamp DNA Maxi kit (QIAGEN) according to the manufacturer’s protocols. Coding exons were captured using the SureSelect XT AUTO HUMAN ALL Exon V5 kit (Agilent Technologies Inc., Santa Clara, CA, USA) and sequenced using the HiSeq2500 system (Illumina Inc., San Diego, CA, USA) in 101 bp paired-end reading mode. The reads were aligned to GRCh37/hg19 using Novoalign (Novocraft Technologies, Selangor, Malaysia) and duplicate reads that were excluded from the analysis were marked using the Novosort software (Novocraft Technologies). Local realignment and variant calling were performed using the HaplotypeCaller in the Genome Analysis Toolkit (Broad Institute, Cambridge, MA, USA) to generate variant call format (VCF) files. Variants found in the cholesteatoma but not in the blood of a participant were considered cholesteatoma-specific variants (CSVs). Variants found in any of the five blood samples were excluded as noise, even if they were CSVs in pair comparisons. CSVs were also identified using MuTect2 and were confirmed using MiSeq after specific polymerase chain reaction amplification. CSVs and six additional genes (*TP63*, *RARA*, *RARB*, *RARG*, *BMP4*, and *TP53*) that are frequently mutated in head and neck tumors were sequenced in another 17 cholesteatoma-blood pair test samples. Bait oligos (Sure Design; Agilent Technologies) were used to capture the exons in target genes. Sequencing was performed using the MiSeq platform and the variants were detected using MuTect2. Except for attic cholesteatomas (n = 14), there were less than three cases of all other types of the pathology (Table 1). Therefore, the correlations between gene mutations and clinical severity, including the presence or absence of bone destruction and granulation tissue formation, were examined using Pearson’s chi-square test only in attic cholesteatomas. The presence or absence of bone destruction was evaluated based on surgical records. At our institution, surgeons always describe the following findings in the operative note: ossicle destruction, bone defects in the facial nerve canal, labyrinthine fistulae, dura exposure, and granulation tissue around the surface of cholesteatoma. The first four findings indicate the presence or absence of cholesteatoma-induced bony destruction.

### Electronic supplementary material

Below is the link to the electronic supplementary material.

Supplementary Material 1

Supplementary Material 2

## Data Availability

Sequence data that support the findings of this study have been deposited in the ClinVar with the accession code SCV003762181 - SCV003762188 (https://www.ncbi.nlm.nih.gov/clinvar/submitters/508279).

## References

[1] Olszewska E (2004). Etiopathogenesis of cholesteatoma. Eur Arch Oto-Rhino-Laryngology.

[2] Alkhaldi AS, Alwabili M, Albilasi T, Almuhanna K (2022). Bezold’s abscess: a case report and review of cases over 20 years. Cureus.

[3] Rüedi L (1979). Pathogenesis and surgical treatment of the middle ear cholesteatoma. Acta Otolaryngol Suppl.

[4] Sadé J (1971). Cellular differentiation of the Middle ear lining. Ann Otol Rhinol Laryngol.

[5] Wang NJ et al. Loss-of-function mutations in Notch receptors in cutaneous and lung squamous cell carcinoma. *Proc. Natl. Acad. Sci. U. S. A* 108, 17761–6 (2011). 10.1073/pnas.1114669108 PMC3203814 22006338

[6] Kuo CL (2015). Etiopathogenesis of acquired cholesteatoma: prominent theories and recent advances in biomolecular research. Laryngoscope.

[7] Jennings BA, Prinsley P, Philpott C, Willis G, Bhutta MF (2018). The genetics of cholesteatoma. A systematic review using narrative synthesis. Clin Otolaryngol.

[8] Bassiouny M, Badour N, Omran A, Osama H (2012). Histopathological and immunohistochemical characteristics of acquired cholesteatoma in children and adults. Egypt J Ear Nose Throat Allied Sci.

[9] Bujia J, Sudhoff H, Holly A, Hildmann H, Kastenbauer E (1996). Immunohistochemical detection of proliferating cell nuclear antigen in middle ear cholesteatoma. Eur Arch Otorhinolaryngol.

[10] Bujía J (1996). Identification of proliferating keratinocytes in middle ear Cholesteatoma using the monoclonal antibody Ki-67. ORL.

[11] de Klerk DrakskogC, Westerberg N, Mäki-Torkko J, Georén E, Cardell SK (2020). Extensive qPCR analysis reveals altered gene expression in middle ear mucosa from cholesteatoma patients. PLoS ONE.

[12] Ozturk K, Yildirim MS, Acar H, Cenik Z, Keles B (2006). Evaluation of c-*MYC* status in primary acquired cholesteatoma by using fluorescence in situ hybridization technique. Otol Neurotol.

[13] Macias JD, Gerkin RD, Locke D, Macias MP (2013). Differential Gene expression in Cholesteatoma by DNA Chip Analysis. Laryngoscope.

[14] Palkó E et al. The c-*MYC* Protooncogene Expression in Cholesteatoma. *Biomed Res. Int* 2014, 1–6 (2014). 10.1155/2014/639896 PMC3934790 24683550

[15] Fukuda A (2021). Notch Signaling in Acquired Middle ear Cholesteatoma. Otol Neurotol.

[16] Aster JC, Pear WS, Blacklow SC (2008). Notch signaling in leukemia. Annu Rev Pathol.

[17] Rebay I (1991). Specific EGF repeats of Notch mediate interactions with Delta and Serrate: implications for Notch as a multifunctional receptor. Cell.

[18] Lawrence N, Klein T, Brennan K (2000). Martinez Arias, A. Structural requirements for notch signalling with delta and serrate during the development and patterning of the wing disc of Drosophila. Development.

[19] de Celis JF, Barrio R, del Arco A, García-Bellido A (1993). Genetic and molecular characterization of a notch mutation in its Delta- and serrate-binding domain in Drosophila. Proc Natl Acad Sci U S A.

[20] Shimizu K (1999). Mouse jagged1 physically interacts with notch2 and other notch receptors. Assessment by quantitative methods. J Biol Chem.

[21] Martincorena I (2018). Somatic mutant clones colonize the human esophagus with age. Science.

