# Supplementary material for: Proto-oncogene mutations in middle ear cholesteatoma contribute to its pathogenesis
Source: BMC Med Genomics. 2023 Nov 15;16:288. doi: 10.1186/s12920-023-01640-6 (PMC10647096; doi:10.1186/s12920-023-01640-6)
Supplement: Supplementary file 1 — Supplementary Material 1 [file 12920_2023_1640_MOESM1_ESM.docx]

Supplemental Figure

Figure 1

Representative image of cholesteatoma with (a) and without (b) granulation tissue of left ear. Image a: granulation tissue is observed around the cholesteatoma (arrow). Image b: only the white cholesteatoma epithelium is observed (arrow). Representative image with (c) and without (d) more than 50% destruction of the ossicles due to cholesteatoma. Image c: destruction of the ossicle (incus) is observed (arrow). Image d: minimal bone resorption is observed (arrow).

The observation regarding the presence or absence of granulation tissue is recorded in the operative note described by surgeon.

Figure 2

Immunohistochemical analysis of *NOTCH1* was performed on a surgical sample (the case not shown in Table I). In this sample, the expression level of *NOTCH1* (2-a) and its downstream target *HES1* (2-b) in the basal cell of cholesteatoma epithelium were found to be weaker compared to postauricular skin. These findings indicate a potential correlation between the function of *NOTCH1* and the pathology of cholesteatoma.
